# Traumatic brain injury-induced submissive behavior in rats: link to depression and anxiety

**DOI:** 10.1038/s41398-022-01991-1

**Published:** 2022-06-07

**Authors:** Matthew Boyko, Benjamin F. Gruenbaum, Ilan Shelef, Vladislav Zvenigorodsky, Olena Severynovska, Yair Binyamin, Boris Knyazer, Amit Frenkel, Dmitry Frank, Alexander Zlotnik

**Affiliations:** 1 Department of Anesthesiology and Critical Care, Soroka University Medical Center and the Faculty of Health Sciences, Ben-Gurion University of the Negev, Beersheba, Israel; 2 Department of Anesthesiology and Perioperative Medicine, Mayo Clinic, Jacksonville, FL USA; 3 Department of Radiology, Soroka University Medical Center and the Faculty of Health Sciences, Ben-Gurion University of the Negev, Beersheba, Israel; 4 Department of Biochemistry and Physiology of the Faculty of Biology and Ecology Oles Gonchar of the Dnipro National University, Dnipro, Ukraine; 5 Department of Ophthalmology, Soroka University Medical Center and the Faculty of Health Sciences, Ben-Gurion University of the Negev, Beersheba, Israel

**Keywords:** Psychology, Neuroscience

## Abstract

Traumatic brain injury (TBI) affects millions of people worldwide, many of whom are affected with post-TBI mood disorders or behavioral changes, including aggression or social withdrawal. Diminished functionality can persist for decades after TBI and delay rehabilitation and resumption of employment. It has been established that there is a relationship between these mental disorders and brain injury. However, the etiology and causal relationships behind these conditions are poorly understood. Rodent models provide a helpful tool for researching mood disorders and social impairment due to their natural tendencies to form social hierarchies. Here, we present a rat model of mental complications after TBI using a suite of behavioral tests to examine the causal relationships between changes in social behavior, including aggressive, hierarchical, depressive, and anxious behavior. For this purpose, we used multivariate analysis to identify causal relationships between the above post-TBI psychiatric sequelae. We performed statistical analysis using principal component analysis, discriminant analysis, and correlation analysis, and built a model to predict dominant-submissive behavior based on the behavioral tests. This model displayed a predictive accuracy of 93.3% for determining dominant-submissive behavior in experimental groups. Machine learning algorithms determined that in rats, aggression is not a principal prognostic factor for dominant-submissive behavior. Alternatively, dominant-submissive behavior is determined solely by the rats’ depressive-anxious state and exploratory activity. We expect the causal approach used in this study will guide future studies into mood conditions and behavioral changes following TBI.

## Introduction

Traumatic brain injury (TBI) is a serious illness associated with the enormous economic burden. Significant economic resources for patients individually and within the healthcare system as a whole are spent on treating both the trauma itself and its consequences, including life-long disability. Approximately 5.3 million people suffer from long-term disabilities as a result of TBI in the United States alone [1].

Survivors of TBI are at increased risk for the development of severe, long-term psychiatric disorders. Prevalence of any psychiatric illness in the first year after the injury has been observed at a rate of 49% following moderate to severe TBI and 34% following mild TBI, compared to 18% in those without TBI [2]. TBI sufferers are particularly susceptible to major depression [3, 4], generalized anxiety disorder [5], post-traumatic stress disorder [2, 6], social withdrawal [7], apathy [7, 8], or aggression [9, 10]. These conditions can persist for decades after brain injury [7, 11] and delay rehabilitation and resumption of employment [12, 13].

Depression is an especially common psychiatric sequelae in patients with TBI [3, 4]. The prevalence of depression ranges from 10–77% [14–16]. Due to its detrimental effects on health, productivity, and quality of life, depression has a significant impact on sufferers. Even after controlling for medical, demographic, and neuropsychological factors, studies have found that depression following TBI is associated with global outcomes [17], a negative impact on social functioning [18], and lower health-related quality of life [19].

Anxiety is another psychiatric condition frequently associated with TBI, at a rate of up to 70% [5]. The condition can persist for many years following TBI [20] and is an increasing burden on global healthcare [21]. Anxiety that continues ten years post-TBI has been found to be a strong predictor of poor psychosocial function [22, 23]. Findings by Mayou et al. suggest that anxiety is a particularly disabling condition for people after TBI [24]. While the pathways associated with the development of specific types of anxiety are not fully understood, the presence of secondary anxiety disorders is associated with a greater impairment that requires a longer recovery period.

Behavioral changes following TBI are reported at rates of 25–88% in people with moderate or severe TBI, with higher prevalence associated with more severe TBI [25–28]. These changes in emotional and social behavior can include indifference, egocentric behavior, emotional lability, poor social judgement and communication, aggression, apathy, impulsive, disinhibited or irritable behavior [29, 30]. Many people with TBI who suffer from these behavioral changes face challenges in integrating back to the workplace or maintaining meaningful social relationships [7, 31–37].

Among the neurobehavioral problems that occur after TBI, aggression is particularly difficult to treat. Studies report a variety of rates of post-TBI aggressive behavior, ranging between 11% [38] and 71% [39]. Like depression and anxiety, aggression has a negative effect on the quality of life of patients, their families, and their caregivers. Despite both pharmacological and non-pharmacological methods that attempt to control this condition, intervention often fails [39].

Another common neurobehavioral effect after TBI is apathy, with estimates on its prevalence varying from 20% [40] to 71% [41], which can impair cognitive function, psychosocial outcome, and rehabilitation efforts. Apathy presents as both a sign and a symptom, and may be considered a diagnosis by itself, in addition to a secondary condition from another underlying condition [42]. There is no discernible association between the appearance of apathy and severity of the brain injury, time since injury, age at injury, or years of education [8].

Aggression, apathy, and other social conditions can be reflected through models of animals that live in groups. In the wild, these animals form social hierarchies to ensure the group’s survival [43, 44]. The approaches by these animals to assert social rank and express dominance and subordination have been well studied in a variety of disciplines [45]. This research has determined that submissive behavior can inhibit aggression and assist in ending disputes before they escalate into violence. Subordination and submission, in addition to the avoidance of inferiority and submission, are associated with anxiety and depression. Models of dominant and submissive behavior have been supported as methods in both human and animal research through self-reporting, observational and behavioral techniques, as well as natural and experimental approaches [46, 47].

However, despite evidence that links anxiety [7, 48], depression [8, 49, 50], aggression [7, 48], and apathy [8] with changes in hierarchical behavior, there have been no studies in the scientific literature on these relationships in the context of TBI. The etiology of these disorders and behavioral changes remains unclear [51]. It is not well-understood, for example, whether psychiatric disorders after TBI are a cause or a consequence of limited functionality [9, 16, 52–55]. Research has focused primarily on cognitive functions such as memory, processing speed, or attention, as possible predictors of the outcome because impairments in cognitive functions occur very frequently following TBI. However, associations between impairments in cognitive function and social outcome and behavior following TBI are fairly weak [53]. Other models of psychosocial outcome following TBI describe neuropsychological deficits as factors that directly contribute to post-injury behavior [56, 57].

Discovery of the correlated and predictive factors leading to aggression and other social behaviors would impart critical information in the prevention and treatment of post-TBI neurobehavioral conditions, especially for improving the efficacy of rehabilitation shortly after TBI when it is most necessary. An effective way to understand the causal relationship between anxiety after TBI, depression after TBI, and changes in social behavior after TBI is to use multivariate statistical methods to analyze behavior.

Due to ethical considerations, it is very difficult to establish a causal relationship in the human population. Therefore, preclinical studies using laboratory animals provide a useful solution. In this study, we use a rat model of TBI to study the sequelae of TBI, especially changes in behaviors relating to mood and socialization.

Similar to the high rates of depression and anxiety in people who suffer from TBI, rodent models of TBI have also shown increased depressive-like and anxiety-like behavior [58]. Rats and mice have a wide expression of social behaviors that can be objectively measured. We are not aware of any animal study that has examined the relationship between anxiety, depression, and social behavior after TBI with multi-factor analysis design. A study on this topic would have important implications for the treatment of anxiety, depression, social changes, and functional limitations following TBI. The principal aim of this manuscript was to test the hypothesis that TBI induces changes in social behavior, particularly dominant-submissive behavior. The second goal of our study was to investigate the relationships between anxiety, depression, and social behavior using statistical analysis of behavioral tests.

## Materials and methods

### Animals

The experiments were conducted in accordance with the recommendation of the Declarations of Helsinki and Tokyo and the Guidelines for the use of Experimental Animals of the European Community. The experiments were approved by the Animal Care Committee of Ben-Gurion University of Negev, Israel. A total of 77 Sprague-Dawley rats (Harlan Laboratories, Israel) were used in this experiment (see Table 1). All rats weighed between 280 and 320 g. Purina Chow and water were made available ad libitum. The temperature in the room was maintained at 22 °C, with a 12 h light–dark cycle. All the tests were conducted in the dark phase between 8 a.m. and 4 p.m.

**Table 1:**
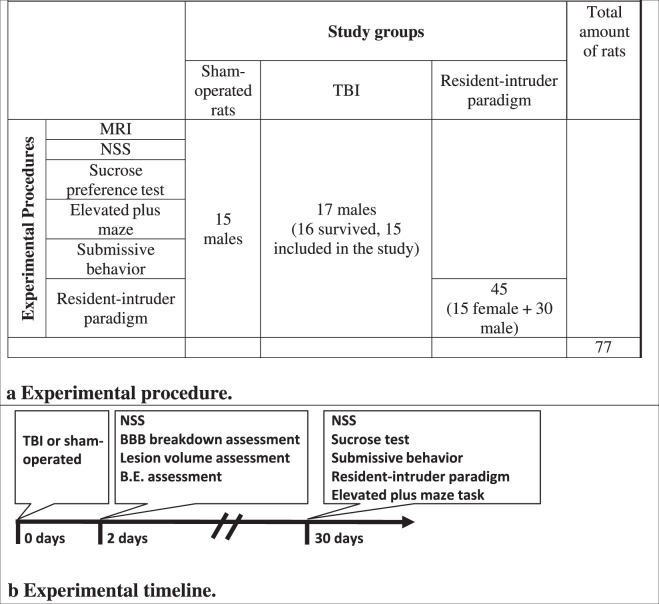
Experimental (A) procedure and (B) timeline.

### Experimental design

Seventy-seven rats were divided into three main groups. Seventeen male rats underwent TBI, fifteen male rats were used as a sham-operated control, and forty-five naïve male and female rats (15 female and 30 male) underwent a resident-intruder paradigm (see Table 1). Rats who died or still had neurological deficits after 4 weeks were excluded from the study in order to avoid the effect of a motor deficit on behavioral performance. Magnetic resonance imaging (MRI) was performed 48 h after intervention. Neurological status was tested at 48 h and 1 month following intervention. All rats from each experimental group underwent a series of behavioral tests at 1 month following intervention (see Table 1).

### Traumatic brain injury (TBI)

TBI was performed as previously described [59]. Rats were anesthetized with 5% inhaled isoflurane; the injury was then affected by a pressure pulse of 2.2 atmospheres. TBI was induced by a fluid-percussion device over 21–23 ms through the 3-way stopcock. Rats in the sham-operated control groups underwent the same procedure but without the administration of the fluid pulse. For a complete protocol for the induction of TBI, see Supplement 1.

### Neurological severity score (NSS)

Two blinded observers calculated NSS as previously described [59]. Points were assigned for motor function and behavioral changes for an overall score between 0, indicating an intact neurological state, and 25, representing the highest neurological impairment (see Supplement 1 for more details).

### Magnetic resonance imaging (MRI)

MRI was used for the determination of the blood–brain barrier (BBB) breakdown (volume transfer constant - K_trans_), DWI, and T2 at 48 h following TBI, as described previously [59]. Measurements were performed in the injured hemispheres and in the symmetric area of the contralateral hemisphere in the penumbra area in close proximity to the necrotic core. A 3 T MRI was used (Ingenia, Philips Medical Systems, Best, The Netherlands) using an eight-channel receive-only coil. The Intellispace Portal workstation (V5.0.0.20030, Philips Medical Systems, Best, The Netherlands) was used for the post-processing of the permeability and perfusion studies. For a complete technical MRI protocol, see Supplement 1.

### MRI analysis

Image analysis was performed by an expert, who was blind to the group assignments. Quantitative ADC maps, in units of square millimeters per second, were generated by the Philips software package (Ingenia, Philips Medical Systems, Best, The Netherlands) and subsequently analyzed using ImageJ software (version 1.50i, National Institutes of Health, Bethesda, Maryland), as previously described [60]. These thresholds were used to identify all pixels’ ADC characteristics on each slice. The viability thresholds were 0.53 × 10-3mm2/s for ADC images [61]. Calculation of lesion volume was performed by the RICH method. The calculation of the lesion volume with the correction for tissue swelling was done using the following formula[60]:

$${\rm{Corrected}}\;{\rm{lesion}}\;{\rm{volume}} = \displaystyle\frac{{{\rm{Lesion}}\;{\rm{volume}} \times {\rm{Contralateral}}\;{\rm{hemisphere}}\;{\rm{size}}}}{{{\rm{Ipsilateral}}\;{\rm{hemisphere}}\;{\rm{size}}}}$$

The calculation of brain edema was done by comparing the contralateral and ipsilateral hemispheres, and performed using the following formula:[62]

$${\rm{Brain}}\;{\rm{edema}} = \displaystyle\frac{{{\rm{Volume}}\;{\rm{of}}\;{\rm{the}}\;{\rm{right}}\;{\rm{hemisphere}} \,-\, {\rm{Volume}}\;{\rm{of}}\;{\rm{the}}\;{\rm{left}}\;{\rm{hemisphere}}}}{{{\rm{Volume}}\;{\rm{of}}\;{\rm{the}}\;{\rm{left}}\;{\rm{hemisphere}}}}$$

### Sucrose preference test

The sucrose preference test was performed as described previously as a method to evaluate anhedonia, which reflects depressive-like symptoms, in a rodent model [63]. Two bottles of sucrose solution were placed in each rat’s cage, consisting of 1% (w/v) solution. Similarly, one of the bottles was replaced by water for 24 h so that the rat could adjust to having one bottle of water and one bottle of sucrose. After this habituation, the rats were deprived of food and water for 12 h. The rats were housed in individual cages with free access to two bottles, one with 100 ml of sucrose solution (1% w/v) and the other with 100 ml of water, for 4 h. After this period, the volume (ml) of the consumed sucrose solution and water was recorded. Sucrose preference was calculated as sucrose preference (%)=sucrose consumption (ml)/(sucrose consumption (ml)+water consumption(ml)) × 100% [63].

### Elevated plus maze task

The plus maze was situated in a dark room and consisted of two open and two closed arms (each with the dimensions 16 × 46 cm). It was constructed from black plastic and positioned 100 cm above the floor. The closed arms, opposite to one another, had a surrounding wall of height 40 cm. 10% ethanol was used to clean the maze prior to the introduction of each animal. Rats were tested on the maze in a randomized order. Each rat was placed in the center of the plus maze facing one of the open arms, and the rat’s behavior was videotaped for 5 min for future analysis. The number of entries into the various arms and time spent in arms of the elevated plus maze was recorded with a video camera (Logitech_HD_Pro_Webcam_C920) and subsequently analyzed using Ethovision XT software (Noldus, Wageningen, Netherlands) [64].

### Dominant-submissive behavior

Seven days before testing, the rats were randomly divided into cages. Each cage contained 1 sham-operated and one TBI rat. Two days before testing, the rats were acclimated to the device with one 15-minute session every day. The apparatus consisted of two transparent Plexiglas boxes (30 cm × 20 cm × 20 cm) connected by a narrow passage (15 cm × 15 cm × 60 cm) [65]. A feeder containing sweetened milk was placed in the middle of the passage [47, 65]. Only one rat was able to fit in the feeder area at a time. During the testing period, the rats only received food in the apparatus. Paired rats were placed an equal distance from the feeder, and their behavior was filmed for five minutes.

The time that each rat spent at the feeder and the first rat to arrive at the feeder were scored afterwards by analyzing recorded video. Although testing occurred over 5 days, the rats were not hungry enough to participate in the experiment properly until the fifth day of testing. Data was measured only on the fifth day.

### Resident-intruder paradigm

The resident-intruder paradigm, a standardized test for aggression, violence and social stress, was performed as previously described [65, 66]. Behavioral analysis was performed by an expert who was blinded to the group assignments. TBI and sham-operated male rats were housed with naïve companion females that were not siblings. They were housed in a polycarbonate cage with a floor space of approximately half a square meter to which they were habituated for 7 days prior to testing with ad libitum food and water. Bedding was not changed during that week or during testing.

One hour before the test, the female companions were removed from the cage, then an hour later, an unfamiliar naive male was placed in the cage with the original permanent male. The interactions of the two rats were recorded for 10 minutes, including the duration and frequency of behavioral parameters. Rats were rated on the following behaviors: chase, upright posture, lateral threat, keep down, start time of first attack, clinch attack, move towards, ano-genital sniffing, social exploration, and non-social exploration. Following testing, the male intruder was removed from the cage and the original male resident was reunited with its original companion female.

### Statistical analysis

Statistical analysis was performed with the SPSS 22 package. A Kolmogorov–Smirnov test was used to decide the appropriate test for the comparisons between the different parameters. The significance of comparisons between groups was determined using the Mann–Whitney, 2-sided (for non-parametric data) and *t* test, 2-sided (for parametric data). The number of rats who came first to the feeder in the dominant-submissive behavior test and mortality rate was analyzed with a chi-square, Fisher’s exact test, 2-sided. To study the correlations between variables and to build a model for predicting submissive behavior, we first performed univariate analysis using Mann–Whitney *U* test, 2-sided (for non-parametric data) or *t* test, 2-sided (for parametric data). Variables with a p-value ≤ 0.05 in the univariate analyses were included in the multivariate model. Potential predictors that differed between the 2 study groups were analyzed by a stepwise discriminant function analysis (DFA). Wilks’ lambda criteria *F*-values for entry was 3.84 and the maximum value prior to removal was 2.71. Normally distributed data and continuous variables were presented as an average ± SD. Non-parametric data were presented as a median ± inner quartile range. Results were considered statistically significant when *p* < 0.05.

## Results

### Mortality

One rat did not survive in the TBI group, and all rats survived in the sham-operated group. The mortality rate was not significantly different between these two groups (6.25% vs 0%, chi-square and Fisher’s exact test). Of note, one additional rat in the TBI group was excluded from the behavioral tests due to incomplete neurological rehabilitation 4 weeks after TBI. Thus, the final number of rats in both the TBI and sham-operated groups were 15 (Table 1).

### Neurological performance

NSS was compared between the experimental groups at 48 h and 1 month after the intervention. The sham-operated group did not show any neurological deficit at 48 h after TBI (0). Compared to sham-operated rats, the NSS at 48 h was significantly greater in TBI rats (6(5–7) vs. 0(0-0), *U* = 0, *p* < 0.01, *r* = 0.89). The data are measured as a count and expressed as median and 25–75 percentile range. At 1 month, there were no differences in neurological performance between the experimental groups (Mann–Whitney U test).

### MRI-determined brain injury

**Fig. 1:**
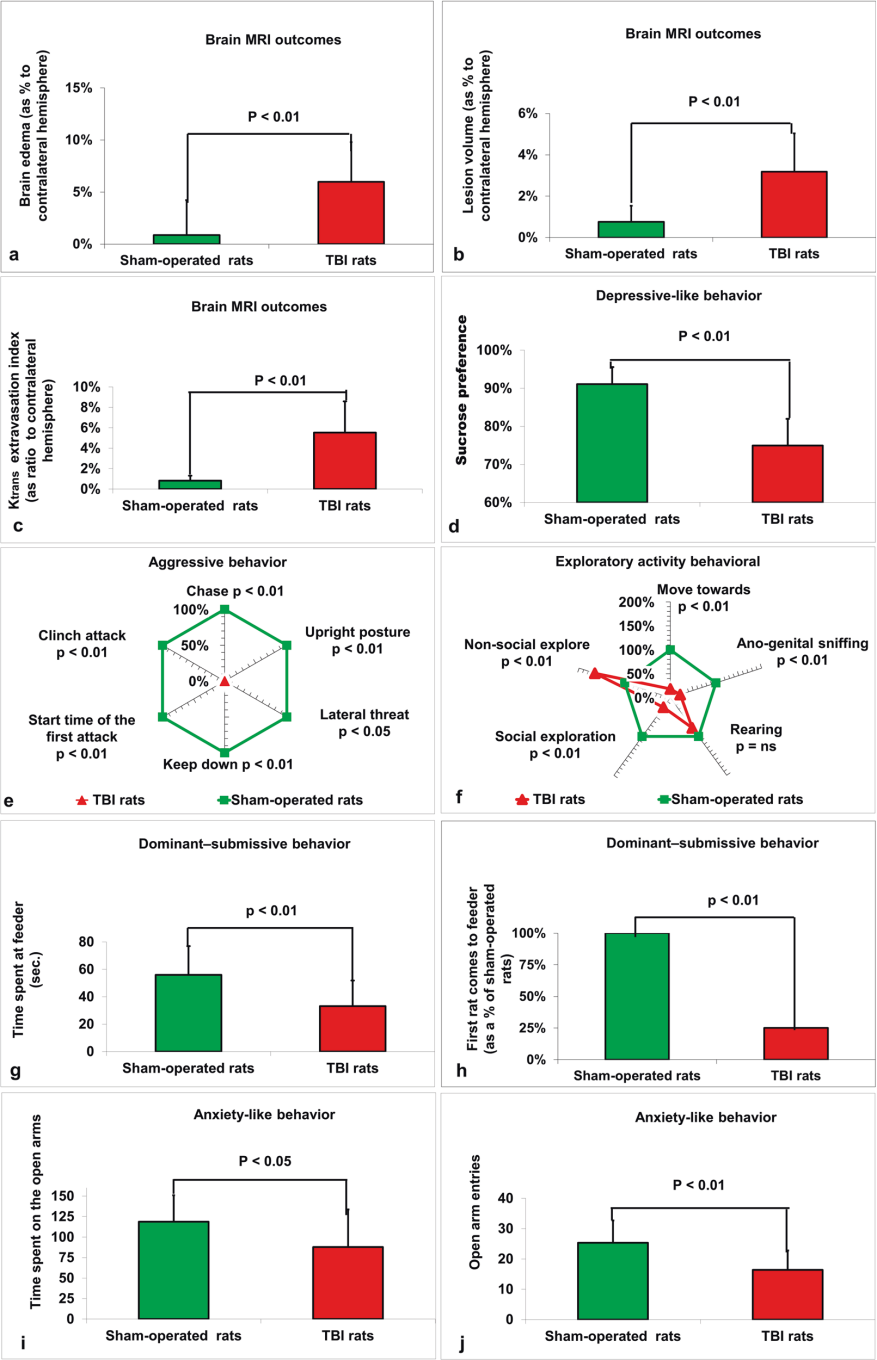
Outcomes following TBI compared to sham-operated rats. **a** MRI-determined brain edema. The data are expressed as a percentage of the contralateral hemisphere and presented as mean ± SD. **b** MRI-determined lesion volume. The data are expressed as a percentage of the contralateral hemisphere and presented as mean ± SD. **c** MRI-determined blood–brain barrier (BBB) breakdown. The data are expressed as a ratio of the contralateral hemisphere and presented as mean ± SD. **d** Sucrose preference. **e** Aggressive behavior. **f** Explorative activity. **g** Time spent at the feeder on the dominant-submissive task. **h** First rat that comes to the feeder on the dominant-submissive task. **i** Time spent on the open arms on elevated plus maze. **j** Open arm entries on elevated plus maze. Data were measured in seconds or count, and presented as mean ± SD, except for in (**e**), (**f**), and (**h**), where data were presented as a percentage.

Forty-eight after intervention, rats that received TBI had significantly greater measurements of brain edema (5.6 ± 3.8% vs. 0.87 ± 3.3%, *t*(28)=−3.7, *p* < 0.01, *d* = 1.33, Fig. 1a), lesion volume (3.2 ± 1.9% vs. 0.76 ± 0.78%, *t*(28) = −4.6, *p* < 0.01, *d* = 1.68, Fig. 1b), and BBB-breakdown determined by Ktrans (5.5 ± 3.1% vs. 0.83 ± 0.48%, *t*(28)=−5.9, *p* < 0.01, *d* = 2.11, Fig. 1c), compared to the sham-operated control rats. Statistics were performed via a Student’s *t* test and the data are expressed as a mean percentage or ratio of the contralateral hemisphere ± SD.

### Sucrose preference

Rats following TBI had significantly lower sucrose preference compared to sham-operated rats (75 ± 7% vs. 91.1 ± 4.5%, *t*(28)=7.5, *p* < 0.001, *d* = 2.74, Student’s *t* test, see Fig. 1d).

### Resident-intruder paradigm

For aggressive behaviors (see Fig. 1e), a Mann–Whitney *U* test showed a statistically significant difference between TBI and sham-operated groups for chase (0 s ± 0 s vs. 2.85 s ± 3.54 s, *U* = 45, *p* < 0.01, *r* =0.63), upright posture (0 counts ± 0 counts vs. 3.8 counts ± 2.37 counts, *U* = 15, *p* < 0.01, *r* = 0.82), lateral threat (0 counts ± 0 counts vs. 7.92 counts ± 18 counts, *U* = 82.5, *p* < 0.05, *r* = 0.38), keep down (0 s ± 0 s vs. 8.54 s ± 10.95 s, *U* = 30, *p* < 0.01, *r* = 0.723), start time of the first attack (0 s ± 0 s vs. 81.66 s ± 210.45 s, *U* = 0, *p* < 0.01, *r* = 0.91), and clinch attack (0 s ± 0 s vs. 102.8 s ± 76.94 s, *U* = 15, *p* < 0.01, *r* = 0.82).

For exploratory behaviors (see Fig. 1f), a Mann–Whitney *U* test showed a statistically significant difference between TBI and sham-operated groups for move towards (7.36 s ± 2.49 s vs. 40.1 s ± 12.99 s, *U* = 0, *p* < 0.01, *r* = 0.85), ano-genital sniffing (2.3 s ± 7.87 s vs. 10.66 s ± 12.16 s, U=40, p < 0.01, r=0.56), social exploration (4.24 s ± 4.96 s vs. 17.34 s ± 13.52 s, *U* = 25, *p* < 0.01, *r* = 0.66), and non-social exploration (450.98 s ± 46.98 s vs. 272.16 s ± 64.96 s, *U* = 0, *p* < 0.01, *r* = 0.85). There was no statistical difference in rearing between the TBI and sham-operated groups (96.42 s ± 37.28 s vs. 124.03 s ± 67.53 s, *U* = 88, *p* = ns, *r* = 0.19).

A Mann–Whitney *U* test did not show any significant difference in rest or inactivity between the TBI and sham-operated groups (38.7 s ± 35.91 s. vs. 21.52 s ± 21.89 s, *U* = 85, *p* = ns, *r* = 0.28).

### Assessment of dominant-submissive behavior

An independent samples *t* test indicated that for the dominant–submissive behavior test, TBI rats spent significantly less time at the feeder than sham-operated controls (33.1 s ± 8.7 s vs. 55.9 s ± 21 s, *t*(28) = 3.14, *p* < 0.01, *d* = 1.15, see Fig. 1 g). The number of rats who came first to the feeder in the dominant-submissive behavior test was significantly lower for TBI rats (3 out of 15) than for control rats (12 out of 15) (*p* < 0.01, according to a chi-square, Fisher’s exact test, see Fig. 1h).

### Elevated plus maze task

A Student’s *t* test showed that there was a statistically significant difference between TBI rats and sham-operated rats in time spent in the open arms (87.82 s ± 45.96 s vs. 118.75 s ± 32.24 s, *t*(28)=2.06, *p* < 0.05, *d* = 0.78, see Fig. 1i) and open arm entries (16.4 ± 6.4 vs. 25.3 ± 7.4, *t*(28)=3.4, *p* < 0.01, *d* = 1.29, see Fig. 1j).

### Multivariable statistics and correlation analysis

Rats were divided into 2 groups, dominant or submissive, based on whether they were the first rat to arrive at the feeder on the dominant-submissive task. The characteristics for each behavioral outcome for dominant and submissive rats are presented in Table 2a. The results of the principal component analysis are presented in Table 2b (Rotated Component Matrix: Varimax with Kaiser Normalization). To define variables as predictors for dominant or submissive behavior, all predictors with a coefficient value greater than 0.2 were used (Table 2b). DFA was conducted to determine which behavioral tests best discriminated between dominant and submissive behavior. In DFA, we used a “stepwise method” to automatically exclude weakly correlated behavioral variables. This method identified 3 main variables for predicting dominant-submissive behavior. The results are presented in Table 3 and Supplement 2. Three variables (rest or inactivity, sucrose preference, time spent on the open arms) were found to be the variables that best differentiated between dominant and submissive behavior. The three behavioral tests (assessing depression, anxiety and exploratory activity) were found to be able to classify rats as either dominant or submissive with an accuracy of 93.3% following validation (Wilks’ λ = 0.288, *p* < 0.01). The correlational relationships between depressive-like, anxiety-like and social behavior are presented in Table 4.

**Table 2:** Principal Component Analysis based on comparison of behavioral outcome characteristics. (A) Characteristics of the behavioral outcomes for dominant and submissive rats. (B) The results of the principal component analysis.

Variables				Mean and SD			*p*‑value (two-tailed)	Statistics reporting*
				Dominant *n* = 15		Submissive *n* = 15		
(A) *Characteristics of the behavioral outcomes for dominant and submissive rats*								
The social behavior assessments	Aggressive behavior	Upright posture		2.67 ± 2.25		1.13 ± 2.64	*p* = ns	*U* = 66, *r* = 0.39
		Lateral threat		7.92 ± 18.23		0 ± 0	*p* = ns	*U* = 82, *r* = 0.38
		Chase		2.67s ± 3.65 s		0.18 s ± 0.56 s	*p* = ns	*U* = 69, *r* = 0.41
		Clinch attack		85.93 s ± 77.85 s		16.87 s ± 54.45 s	*p* < 0.05	*U* = 46, *r* = 0.56
		Keep down		8.12 s ± 11.24 s		0.42 s ± 0.92 s	*p* < 0.05	*U* = 63, *r* = 0.43
		Start time of the first attack		161 s ± 274 s		520 s ± 210 s	*p* < 0.05	*U* = 39, *r* = 0.48
	Exploratory activity behavioral	Move towards		34.15 s ± 19.13 s		13.31 s ± 12.27 s	*p* < 0.05	*U* = 48, *r* = 0.49
		Ano-genital sniffing		10.72 s ± 12.16 s		2.24 s ± 3.71 s	*p* < 0.05	*U* = 44, *r* = 0.53
		Rearing		120.72 s ± 72.45 s		99.73 s ± 29.55 s	*p*=ns	*t*(28) = 1; *d* = 0.38
		Social exploration		16.61 s ± 13.73 s		4.96 s ± 6.18 s	*p* < 0.05	*t*(28) = 3; *d* = 1.1
		Non-social explore		303 s ± 108 s		420 s ± 68 s	*p* < 0.05	*t*(28) = −3.6; *d* =−1.3
		Rest or inactivity		17.94 s ± 16.21 s		42.29 s ± 36.7 s	*p* < 0.05	*t*(28) = −2.4; *d* = −0.9
	Hierarchical behavior	Time spent at feeder		60.13 s ± 16.7 s		28.93 s ± 16.38 s	*p* < 0.05	*t*(28) = 5.2; *d* = 1.9
Assessment of depressive-like behavior	Sucrose Preference test	Sucrose preference		91 ± 5%		75 ± 7%	*p* < 0.05	*t*(28) = 6.8; *d* = 2.6
Assessment of anxiety-like behavior	Elevated plus maze test	Time spent on the open arms		136 s ± 27 s		70 s ± 28 s	*p* < 0.05	*t*(28) = 6.5; *d* = 2.4
		Open arm entries		25.67 ± 7.69		16.07 ± 6.05	*p* < 0.05	*t*(28) = 3.8; *d* = 1.4
			**Variables**					
(B) *The results of the principal component analysis.*								
Aggressive behavior			Clinch attack		0.813			
			Keep down		0.867			
			Start time of the first attack		−0.812			
Exploratory activity behavioral			Move towards		0.684			
			Ano-genital sniffing					
			Social exploration					
			Non-social explore		−0.695			
			Rest or inactivity		−0.236			
Depressive-like behavior			Sucrose preference		0.530			
Anxiety-like behavior			Open arm entries		0.662			
			Time spent on the open arms		0.302			

For *t* tests, mean and SD, degrees of freedom, statistics *t* and Cohen’s d are presented. For the Mann–Whitney test, the median and range, statistics *U*, and effect size, and *r* = *Z*/√*N* are reported. The original data was measured in sec or count. Rotated Component Matrix: Varimax with Kaiser Normalization. The following tests best describe the predictors within the study group. Coefficients whose values were less than 0.2 are not shown.

**Table 3:** The results of the canonical discriminant function coefficients.

Unstandardized coefficients	Function 1
Rest or inactivity	−0.017
Sucrose preference	8.590
Time spent on the open arms	0.025
(Constant)	−9.215

Three variables were found to be the best at discriminating between dominant vs. submissive behavior: rest or inactivity, sucrose preference, and time spent on the open arms. These three behavioral tests were able to classify rats into the dominant or submissive with an accuracy of 93.3% following validation (Wilks’ λ = 0.288, *p* < 0.01). Coefficients whose values are less than 0.2 were not shown in the table. The “stepwise method” algorithm was used to automatically select predictors. d = −9.215–0.17*” Rest or inactivity” + 8.590* “Sucrose preference” + 0.25*“Time spent on the open arms”.

**Table 4:** The correlational relationships between depressive-like, anxiety-like and social behavior.

Behavioral tests and their variables			Assessment of anxiety-like behavior		Assessment of depressive-like behavior
			Open arm entries	Time spent on the open arms	Sucrose preference test
The social behavior assessments	Aggressive behavior	Upright posture	Rs = 0.615; *p* < 0.01	Rs = 0.491; *p* < 0.01	Rs = 0.65; *p* < 0.01
		Lateral threat	*p* = ns	*p* = ns	*p* = ns
		Chase	*p* = ns	Rs = 0.364; *p* < 0.05	Rs = 0.584; *p* < 0.01
		Clinch attack	Rs = 0.585; *p* < 0.01	Rs = 0.489; *p* < 0.01	Rs = 0.738; *p* < 0.01
		Keep down	Rs = 0.551; *p* < 0.01	Rs = 0.439; *p* < 0.05	Rs = 0.578; *p* < 0.01
		Start time of the first attack	Rs = 0.−581; *p* < 0.01	Rs= −0.55; *p* < 0.01	Rs = −0.792; *p* < 0.01
	Exploratory activity behavioral	Move towards	Rs = 0.628; *p* < 0.01	Rs = 0.499; *p* < 0.01	Rs = 0.785; *p* < 0.01
		Ano-genital sniffing	*p* = ns	*p* = ns	Rs = 0.58; *p* < 0.01
		Rearing	*p* = ns	*p* = ns	*p* = ns
		Social exploration	Rp = 0.427; *p* < 0.05	Rp = 0.552; *p* < 0.01	Rp = 0.56; *p* < 0.01
		Non-social explore	Rp= −0.548; *p* < 0.01	Rp = −0.499; p < 0.01	Rp = −0.73; *p* < 0.01
	Rest or inactivity	Rest or inactivity	*p* = ns	*p* = ns	*p* = ns
	Hierarchical behavior	Time spent at feeder	Rp = 0.398; *p* < 0.05	Rp = 0.52; *p* < 0.01	Rp = −0.667; *p* < 0.01
Assessment of depressive-like behavior	Sucrose preference test	Sucrose preference	Rp = 0.549; *p* < 0.01	Rp = 0.715; *p* < 0.01	

## Discussion

Previous studies have analyzed the relationship between post-TBI anxiety, post-TBI depression and post-TBI poor functional status, and changes in social cognition in humans [52, 67, 68]. It should be noted that due to ethical constraints, however, it can be very difficult to identify a causal relationship in the human population. Preclinical studies using laboratory animals can elucidate the causality of these relationships, though no other animal study has examined the relationship between anxiety, depression and social behavior after TBI using multi-factor analysis design, as we present here.

Translational assessments of any psychiatric disorder are, by their nature, challenging. The diagnostic criteria of these are complex, heterogeneous, non-exclusive, and multi-factorial. In addition, several of the symptoms associated with psychiatric disorders are uniquely human and cannot be accurately translated—for example, auditory hallucinations, verbal aggression, or suicidal tendencies are challenging to assess in non-human subjects. It is also important to recognize that TBI is a highly heterogeneous condition; however, there is a consensus in the scientific literature that, depending on the injury mechanism, the severity of the injury, and time post-injury, can result in a broad range of pathological changes that may manifest in different behavioral deficits [69–72]. Similar to the clinical evidence described earlier, there are many reports which describe increases in depressive/anxiety behavior in models of TBI [69–77]. The most striking feature of these reports is the consistency of the depression [70] / anxiety [23] phenotype, which is commonly observed despite these studies being conducted in many different laboratories and incorporating a wide variety of models of injury, ages, species of animal, time of measurement after injury, injury frequency, injury severity and reported outcomes. This variety suggests that this is indeed a robust consequence of TBI.

In this study, we investigated the changes in social behavior of rats after TBI, particularly its effect on hierarchical behavior. Using principal component analysis and discriminant function analysis, the relationship between post-TBI depression, post-TBI anxiety, and various aspects of social behavior in rats following TBI was also investigated.

As expected, the rats from the TBI group 48 h after the head injury had neurological deficits, increased cerebral edema, and increased lesion volume assessed by MRI compared to sham-operated rats. Assessing neurological symptoms is the most sensitive and popular method used in the model of traumatic brain injury [59]. The specificity of neurological deficit after a stroke and TBI is such that spontaneous recovery occurs within a month after the injury [78, 79]. Since the main goal of our work was to assess associated changes in behavior after brain injury, we chose a model with moderate brain injury, in which spontaneous neurological recovery occurs within a month, so that neurological deficit after head injury does not affect the assessment of behavioral outcome [79].

In TBI rats, we found that depressive- and anxious-like behaviors developed. This phenomenon has been well documented in both human and animal literature including rat models of stroke [50, 61], TBI [13, 20, 77, 80–83], and subarachnoid hemorrhage [84–86].

Changes in social behavior in the human population, including aggression, and functional outcomes after TBI are well documented in the scientific literature [2, 4, 5, 7, 9, 16, 29]. Assessment of aggressive behavior in the experimental groups in our study showed that the level of aggression in TBI rats was reduced compared to the sham-operated rats. The explanation for this phenomenon is that the appearance of aggressive behavior is closely related to the area of injury. Disorders in the amygdala, hypothalamus, and prefrontal cortex are associated with the development of aggressive behavior [87, 88]. A feature of TBI in our model is that brain damage was not directly associated with the amygdala, hypothalamus, and prefrontal cortex, which are involved in the development of aggressive behavior. Thus, we did not register a high level of aggression in injured rats. In contrast, we have documented post-TBI apathetic behavior, which is also a common complication of TBI in humans [8].

The decrease in aggression in TBI rats can also be explained by the influence of depression and anxiety, a correlation that is well documented in earlier scientific studies [9, 48, 89–91]. Because exploratory behavior is related to the level of aggression, as shown in an earlier study in which more aggressive rodents show high levels of exploratory activity [92], it was not surprising that our study found that the exploratory behavior of trauma rats was significantly reduced compared to sham rats. It also correlated with their level of aggression.

Predicting dominant-submissive behavior based on assessing patterns of anxiety, depressive, aggressive, and exploratory behavior using discriminant analysis can explain 93.3% of the variance. In the scatter plot of the two discriminant functions 2 groups are separated nicely. The “stepwise method” eliminated weakly correlated behavioral variables and identified 3 main variables for predicting dominant-submissive behavior. Surprisingly, the machine algorithm chose one variable from each behavioral paradigm: Exploratory activity behavioral (Rest or inactivity), depressive-like behavior (sucrose preference), and anxiety-like behavior (time spent on the open arms). Thus, the machine’s algorithm determined that in rats, aggression is not a principal prognostic factor for dominant-submissive behavior. Instead, dominant-submissive behavior seems to be determined solely by the rats’ depressive-anxious status and exploratory activity.

The main conclusion of this study was that TBI can cause changes in hierarchical status by decreasing or increasing levels of aggression, which, in turn, depends on factors of the site of the head injury and the development of anxiety-depressive patterns of behavior. Based on known behavioral patterns in rats, we built a mathematical model to predict hierarchical relationships in cohorts of sham-operated and TBI-rats with a prediction accuracy of 93.3% (93.3% for dominant behavior and 93.3% for submissive behavior). A correlation analysis was also performed between behavioral outcomes. The results suggest that submissive behavior is associated with a decrease in aggression, which, most likely, as a consequence of the depressive-like or anxiety-like states of rats after TBI, which should be targeted by the main treatment. Similar research in the human population and animal models shows that depression and anxiety are closely related to aggression [49, 91, 93]. In summary, the model presented here helps to clarify the relationship between depression, anxiety, and social behavior following TBI. We anticipate that future studies on the long-term sequelae and related consequences of TBI will consider multivariate analysis to understand causal relationships between TBI, behavior, and mood.

This study has some limitations. We did not study the female population in our study, because the patterns of hierarchy and dominant-submissive relations of female are different from those of men. Studies have not yet established a territorial hierarchy in the female population. Thus, we foresee difficulties in extrapolating the male resident test results to the female population. In addition, this study did not allow for an investigation into the effects of memory and cognition, which have been well studied in the human population. However, there are many studies that have not found or found a very weak correlation between cognitive ability and dominance in the human population. As stated above, associations between impairments in cognitive functions and social outcome and behavior following TBI are fairly weak [53] and good cognitive recovery does not ensure good recovery in social outcomes [53, 94]. In contrast, human clinical studies have shown that depression and anxiety are closely related to social behavior. Therefore, in this study, we focused on studying the changes in social behavior that occur as a result of TBI and the relationship with post-TBI anxiety and post-TBI depression. In future studies, we also hope to analyze the long-term outcomes of TBI on these behavioral outcomes after 6 months.

## Supplementary information

Supplemental material 1

Supplemental material 2

Supplemental material 2 Legend
