# Supplementary material for: Traumatic brain injury-induced submissive behavior in rats: link to depression and anxiety
Source: Transl Psychiatry. 2022 Jun 7;12:239. doi: 10.1038/s41398-022-01991-1 (PMC9174479; doi:10.1038/s41398-022-01991-1)
Supplement: Supplementary file 1 — Supplemental material 1 [file 41398_2022_1991_MOESM1_ESM.docx]

**Supplement 1.** Technical protocol for MRI, complete description of neurological tests and complete protocol for the induction of TBI.

*Traumatic brain injury (TBI)*

TBI was performed as previously described [1]. Rats were anesthetized with 5% inhaled isoflurane for induction and 1.5-2.5% for maintenance, with equal parts medical air and oxygen. Prior to incision, the scalp was infiltrated with 0.5% bupivacaine. It was then perforated and reflected laterally with the left temporal muscle, while the underlying periosteum was dissected to reveal the skull. A craniotomy was performed at 5-mm using a trephine (Roboz Surgical Instrument Co., Gaithersburg, MD) fastened to the drill bit of an electrical drill (Stoelting, Wood Dale, IL). The center of the craniotomy was positioned 4 mm lateral and 4 mm posterior to bregma. A Luer 3-way stopcock was fixed and additionally held in place by cyanoacrylate adhesive and dental acrylic. The injury was then effected by a pressure pulse of 2.2 atmospheres [2, 3]. TBI was induced by a fluid-percussion device over 21-23 msec through the 3-way stopcock. The fluid pulse from the piston plunger, through the pendulum, was enacted via continuous saline fluid into the dura to allow for efficient transmission of the pressure pulse. Rats in the sham-operated control groups underwent the same procedure but without the administration of the fluid pulse.

Rats were monitored by a pulse-oximeter during the surgery to ensure uninterrupted measurements of heart rate and blood oxygen levels. After TBI induction, the incision was sutured, and the rats were allowed to recover from anesthesia.

*Neurological severity score (NSS)*

Two blinded observers calculated NSS as previously described [1]. Points were assigned for motor function and behavioral changes for an overall score between 0, indicating an intact neurological state, and 25, representing highest neurological impairment. Testing included: 1) The rats’ inability to exit a 50 cm diameter circle, when placed in its center. This task is performed three ties, with sessions lasting 30, 60 and more than 60 minutes each. This test has a scale from 0 to 3. 2) The rats’ loss of righting reflex. Here, the rat is placed on its back in the palm of the investigator’s hand. This is scored from 0 (not able to right itself) to 1 (able to right itself). 3) The rats’ inability to resistant forced positioning, or hemiplegia. This test has a scale from 0 to 1. 4) The rats’ reflexive bending of the hindlimb when risen by the tail. This test has a scale from 0 to 2. 5) The rats’ ability to walk straight when placed on the floor. This test has a scale from 0 to 1. 6) The rats’ response to three reflexive behaviors: the pinna reflex, the corneal reflex, and the startle reflex. For the pinna reflex, the investigator gives light tactile stimulation to test ear retraction (scale of 0 to 1). For the corneal reflex, the investigator monitors blink response after a needle is lightly applied to the eye (scale of 0 to 1). For the startle reflex, a pen is dragged across the top of a wire cage (scale of 0 to 1). 7) The rats’ loss of seeking behavior or prostration. This test has a scale from 0 to 1. 8) The rats’ limb reflexes (bilateral forelimbs and hindlimbs). This test has a scale from 0 to 2. 9) The rats’ performance on a beam balance task (1.5cm wide). This test has three sessions of increasing time from 20 seconds, 40 seconds, and more than 60 seconds (scale of 0 to 3). 10) The rats’ performance on a beam walking test with three different beams (8.5 cm, 5 cm, and 2.5 cm wide). This test has a scale from 0 to 3.

*Magnetic resonance imaging (MRI)*

MRI was used for the determination of the blood brain barrier (BBB) breakdown (volume transfer constant - K_trans_), DWI, and T2 at 48 hours following TBI, as described previously [1]. Measurements were performed in the injured hemispheres and in the symmetric area of the contralateral hemisphere in the penumbra area in close proximity to the necrotic core. Animals were maintained under general anesthesia (1.5% isoflurane in oxygen). A tail vein catheter was introduced and connected to a syringe containing a solution of Gadopentetic acid (Gd-DTPA) (Dotarem, 0.5 mmol/ml Guerbet, France). A 3T MRI was used (Ingenia, Philips Medical Systems, Best, The Netherlands) using an eight-channel receive-only coil. Localising T2w turbo spin echo (TSE) sequences were acquired in sagittal and coronal planes with TR/TE=3000/80 msec, turbo factor=15, water-fat shift=1.6 pixels, resolution (freq×phase×slice)=0.47×0.41×2.0mm and one average for a scan time of 1:00 min. In the axial direction the scan parameters were repetition time/echo time (TR/TE)=3000/80 msec, turbo factor=14, water-fat shift=1.6 pixels, resolution (freq×phase×slice)=0.37×0.33×2.0mm. Four averages were acquired for a scan time of 4:54 min. Diffusion tensor imaging in six directions was performed in the axial direction using a multi-shot STimulated Echo Acquisition Mode (STEAM) spin-echo, echo-planar sequence with repetition time/mixing time/echo time (TR/TM/TE)=1355/15.0/143 msec, SENSitivity Encoding (SENSE) reduction factor=1.5, turbo factor=19, b=1000 s/mm2, resolution (freq×phase×slice)=0.55×0.55×2.0mm with spectrally-selective fat suppression. Five signal averages were acquired for a scan time of 8:40 min. T1 permeability studies were performed using a segmented 3D T1w-FFE sequence with 50 dynamics for a total scan time of 25:52 min. The scan parameters were TR/TE=16/4.9 msec, turbo factor=48, SENSE factor 1.5, resolution (freq×phase×slice)=0.30×0.37×2.0mm, tip angle=80 and two signal averages for a scan time of 31 sec/dynamic. Three calibration scans with identical resolution preceded the dynamic sequence with tip angles 50, 100 and 150. The contrast agent was injected after the 5th dynamic scan. T2 perfusion studies were carried out using a dynamic, single-shot gradient-echo epi sequence with spectrally-selective fat suppression. The scan parameters were TR/TE=1300/40 msec, resolution (freq×phase×slice)=0.64×0.69×2.0mm, and one signal average giving a scan time of 1.3sec/dynamic. A total of 150 dynamics were acquired for a scan time of 3:19 min. The Intellispace Portal workstation (V5.0.0.20030, Philips Medical Systems, Best, The Netherlands) was used for the post-processing of the permeability and perfusion studies.

**References**:

1. Frank, D., et al., *A novel histological technique to assess severity of traumatic brain injury in rodents: comparisons to neuroimaging and neurological outcomes.* Frontiers in neuroscience, 2021: p. 1314.

2. Frank, D., et al., *A Metric Test for Assessing Spatial Working Memory in Adult Rats following Traumatic Brain Injury.* Journal of Visualized Experiments: Jove, 2021(171).

3. Kabadi, S.V., et al., *Fluid-percussion–induced traumatic brain injury model in rats.* Nature protocols, 2010. **5**(9): p. 1552-1563.
