# Supplementary figures and images for: Traumatic brain injury-induced submissive behavior in rats: link to depression and anxiety
Source: Transl Psychiatry. 2022 Jun 7;12:239. doi: 10.1038/s41398-022-01991-1 (PMC9174479; doi:10.1038/s41398-022-01991-1)

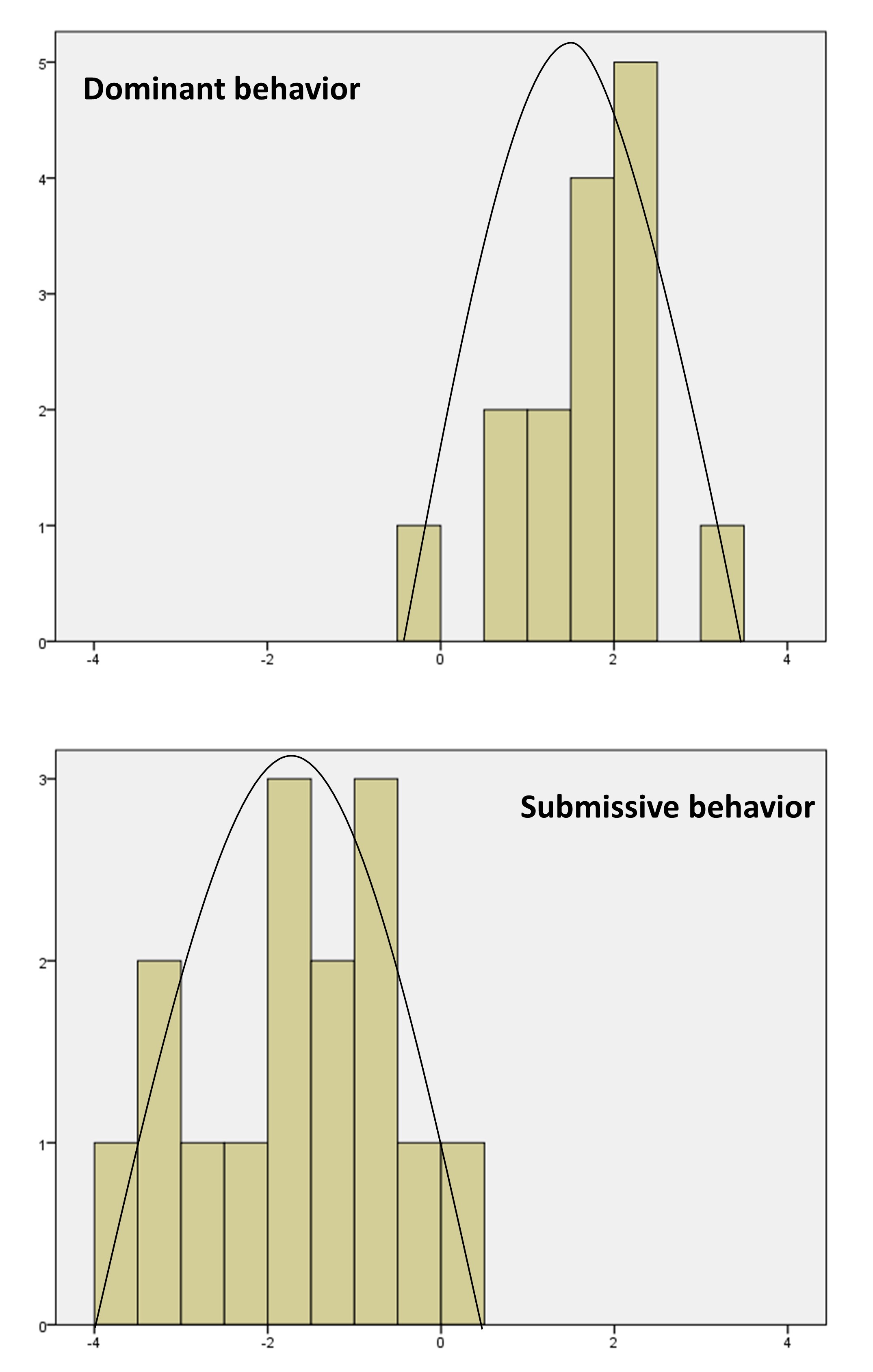

Supplement: Supplementary file 2 — Supplemental material 2 [file 41398_2022_1991_MOESM2_ESM.jpg]
