# Supplementary material for: Traumatic brain injury-induced submissive behavior in rats: link to depression and anxiety
Source: Transl Psychiatry. 2022 Jun 7;12:239. doi: 10.1038/s41398-022-01991-1 (PMC9174479; doi:10.1038/s41398-022-01991-1)
Supplement: Supplementary file 3 — Supplemental material 2 Legend [file 41398_2022_1991_MOESM3_ESM.docx]

**Supplement 2. Histogram of values of discriminant function.** DFA was calculated for dominant-submissive behavior using the 3 predictors: rest or inactivity, sucrose preference, and time spent on the open arms.
